# Nitrate-mediated luminal expansion of *Salmonella* Typhimurium is dependent on the ER stress protein CHOP

**DOI:** 10.1128/mbio.01008-26

**Published:** 2026-06-15

**Authors:** Elizabeth A. Alexander, Lydia A. Sweet, Sharon K. Kuss-Duerkop, Mariana X. Byndloss, Tamarah E. Schaberg, A. Marijke Keestra-Gounder

**Affiliations:** 1 Department of Immunology and Microbiology, University of Colorado Anschutz Medical Campus 129263, Aurora, Colorado, USA; 2 Vanderbilt University Medical Center, Howard Hughes Medical Institute 2405, Nashville, Tennessee, USA; 3 Department of Pathology, Microbiology, and Immunology, Vanderbilt University Medical Center 12328, Nashville, Tennessee, USA; 4 Vanderbilt Institute of Infection, Immunology, and Inflammation, Vanderbilt University Medical Center 12328, Nashville, Tennessee, USA; 5 Vanderbilt Digestive Disease Center, Vanderbilt University Medical Center 12328, Nashville, Tennessee, USA; 6 Vanderbilt Microbiome Innovation Center, Vanderbilt University 5718, Nashville, Tennessee, USA; Stanford University School of Medicine, Stanford, California, USA

**Keywords:** *Salmonella*, innate immunity, CHOP

## Abstract

*Salmonella* Typhimurium (*S*. Typhimurium) is an enteric pathogen that employs a variety of mechanisms to exploit inflammation, resulting in expansion in the intestinal tract, but host factors that contribute to or counteract the luminal expansion are not well-defined. Endoplasmic reticulum (ER) stress induces inflammation and plays an important role in the pathogenesis of infectious diseases. However, little is known about the contribution of ER stress-induced inflammation during *Salmonella* pathogenesis. Here, we demonstrate that the ER stress markers *Hspa5* and *Xbp1* are induced in the colon of *S*. Typhimurium-infected mice, but the pro-apoptotic transcription factor *Ddit3,* which encodes the protein CHOP, is significantly reduced. *S*. Typhimurium-infected mice deficient for CHOP displayed a significant decrease in inflammation, colonization, dissemination, and pathology compared to littermate control mice. Preceding the differences in *S*. Typhimurium colonization, a significant decrease in *Nos2* gene and inducible nitric oxide (NO) synthase (iNOS) protein expression was observed. Deletion of *Chop* decreased the bioavailability of nitrate in the colon, leading to a reduced fitness advantage of wild-type *S*. Typhimurium over a *napA narZ narG* mutant strain (deficient in nitrate respiration). CD11b^+^ CCR2^+^ myeloid cells, but not intestinal epithelial cells, produced iNOS, resulting in nitrate bioavailability for *S*. Typhimurium to expand in the intestinal tract in a CHOP-dependent manner. Altogether, our work demonstrates that the host protein CHOP facilitates iNOS expression in CD11b^+^ CCR2^+^ cells, thereby contributing to luminal expansion of *S*. Typhimurium via nitrate respiration.

**IMPORTANCE:**

*Salmonella* Typhimurium is a gastroenteric bacterium that replicates to large numbers within the gastrointestinal (GI) tract, allowing for efficient host-to-host transmission. One strategy that allows *Salmonella* to expand in the GI tract is via nitrate respiration that is generated during *Salmonella* infections. The host protein CHOP is activated within the unfolded protein response (UPR), an adaptive response pathway that is activated when cells are undergoing endoplasmic reticulum (ER) stress. ER stress has been implicated in several infectious and inflammatory diseases; however, little is known about the contribution of ER stress and the UPR during *Salmonella* infections. Our results presented here provide more insight into the role of CHOP in the production of nitrate and the subsequent growth of *Salmonella* in the GI tract. Altogether, our research provides a better understanding of the contribution of the ER stress protein CHOP in intestinal health and disease.

## INTRODUCTION

*Salmonella enterica* serovar Typhimurium (*S*. Typhimurium) is a gram-negative enteric bacterial pathogen that actively induces intestinal inflammation, which allows *S*. Typhimurium to outcompete the host microbiota and expand within the gastrointestinal (GI) tract (1, 2). The main virulence factors required for *Salmonella* to induce intestinal inflammation are two type III secretion systems (T3SS) (1–4). The T3SS-1 allows *S*. Typhimurium to invade intestinal epithelial cells (IECs), resulting in the production of cytokines/chemokines, including CXCL1 and CCL2, thereby attracting inflammatory phagocytes (macrophages, monocytes, neutrophils, and dendritic cells) to the site of infection (3). The T3SS-2 is required for survival and replication within macrophages (5). In addition to the T3SSs, *Salmonella* induces inflammatory responses via pathogen-associated molecular patterns that are detected by pattern recognition receptors (PRRs) (6, 7). Early innate immune responses during *S*. Typhimurium infection include the production of an array of chemokines and cytokines such as CXCL1, TNFα, IL-1β, and IL-23 (8–10). Intestinal epithelial cells are the predominant producers of neutrophil chemoattractant CXCL1 during *S*. Typhimurium infection (9, 11, 12). TNFα and IL-1β are mainly produced by inflammatory monocytes, and IL-23, produced by dendritic cells, is a crucial cytokine in the development and maintenance of the pro-inflammatory Th17 response (9, 13). Each of these chemokines and cytokines has a distinct expression pattern during acute *Salmonella* infection, with CXCL1 and IL-1β induced early and increasing over time, while TNFα and IL-23 peak in expression at 48 h (14, 15). In addition to the induction of chemokines and cytokines, *S*. Typhimurium also causes robust expression of *Nos2. Nos2* is the gene encoding for inducible nitric oxide (NO) synthase (iNOS) that catalyzes the production of nitric oxide from L-arginine. Nitric oxide radicals react with superoxide radicals, leading to the generation of nitrate (NO_3_^-^). Nitrate enhances the growth of *S*. Typhimurium via nitrate respiration (16, 17). It was demonstrated that different cells in the intestinal tract produce nitrate, but interestingly, only the nitrate produced by phagocytic infiltrates is utilized by *S*. Typhimurium (18, 19).

Upon infection, the intestinal tract must cope with diverse cellular stresses, and as a response, cells activate mechanisms to support cellular functions to adapt to changing environmental conditions. Activation of the unfolded protein response (UPR) is one such mechanism that is induced upon endoplasmic reticulum (ER) stress (20). The ER is a highly dynamic organelle that exerts a major role in coordinating signaling pathways to ensure cellular homeostasis. The ER is the site of synthesis and folding of proteins; however, under different stressful pathological and physiological conditions, the ER is unable to maintain homeostasis and activates the UPR (20). Three transmembrane receptors, ATF6, PERK, and IRE1α, are activated and regulate biological processes such as the inhibition of protein translation, autophagy, and inflammation to restore cellular homeostasis. Under homeostatic conditions, the chaperone BiP, encoded by the *Hspa5* gene, is bound to these receptors, thereby preventing their activation. Perturbation of the ER triggers the release of BiP from ATF6, PERK, and IRE1α, resulting in dimerization and phosphorylation of these receptors to an active state (20). ATF6, PERK, and IRE1α subsequently activate the transcription factors ATF6f, ATF4, and XBP1, respectively, which then bind to ER stress elements that result in the transcription of genes such as *Hspa5*, *Xbp1*, and *Ddit3* (herein referred to as *Chop*), the gene encoding transcription factor CCAAT/enhancer-binding protein homologous protein (CHOP) (Fig. S1) (20). CHOP is a downstream apoptosis-promoting target of the PERK-ATF4 pathway, resulting in ER stress-induced cell death when the ER stress is severe and/or prolonged (21). Although the role of CHOP has focused mostly on the induction of cell death, more recent studies have reported a role for CHOP in regulating inflammation during infections (22, 23). Infection of myeloid cells with *Chlamydia trachomatis* resulted in increased *Chop* expression and binding of CHOP to the *IL23* promoter (23). Increased expression of *Chop* mRNA and CHOP protein was also detected in trophoblasts infected with *Brucella abortus* (24). Activation of *Chop* during *Listeria monocytogenes* infection was shown to be detrimental to the host, leading to increased morbidity and mortality (25). Shiga toxins expressed by enteric pathogens *Shigella dysenteriae* 1 and enterohaemorrhagic *Escherichia coli* (EHEC) increased CHOP expression and induced monocytic cell apoptosis (26). Thus, CHOP has a critical, yet not well-defined, role during bacterial infections.

Although a lot is known about the mechanisms of *Salmonella*-induced intestinal inflammation, not much is known about the contribution of ER stress during *Salmonella* infections. Treatment of *Salmonella*-infected mice with the ER stress inhibitor TUDCA resulted in increased colonization of the small intestine because of decreased lysozyme production mediated by the ER stress-induced secretory autophagy pathway in Paneth cells (27). In HeLa cells, however, it was demonstrated that cells pretreated with the ER stress inducer thapsigargin resulted in increased *Salmonella* replication, suggesting that activation of ER stress provides a favorable environment for *Salmonella* (28). Here, we set out to investigate the involvement of ER stress, and more specifically the function of ER stress-induced CHOP, in the *Salmonella*-induced colitis model as well as the contribution of CHOP to inflammation, colon pathology, and luminal expansion of *S.* Typhimurium.

## RESULTS

### Activation of the ER stress response during *S*. Typhimurium infection

**Fig 1:**
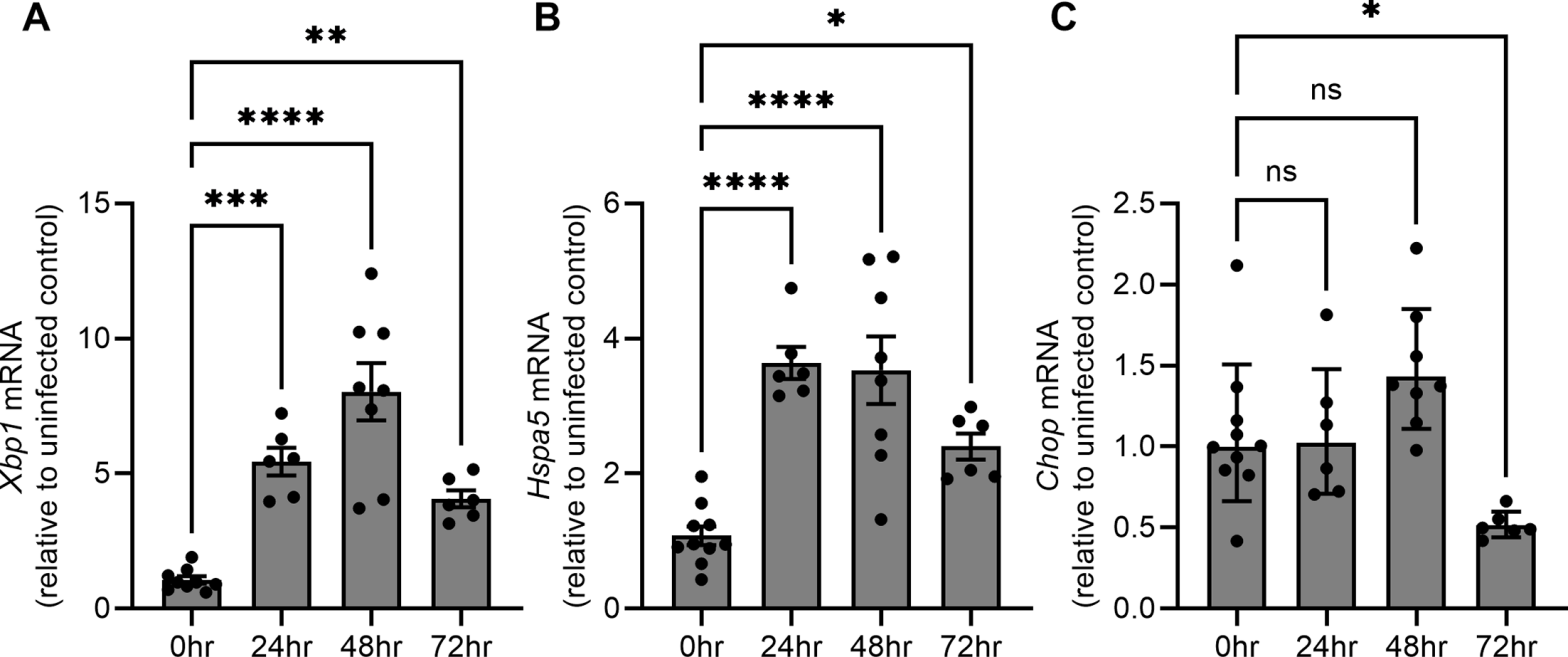
*S.* Typhimurium activates the UPR *in vivo*. RNA was extracted from the colon of *S.* Typhimurium (SL1344) infected (10^8^ cfu/mouse) and uninfected mice (C57BL/6) and analyzed by qRT-PCR for expression of the ER stress markers *Xbp1* (**A**), *Hspa5* (**B**), and *Chop* (**C**). Data shown as mean ± standard error of the mean with 6–10 mice per group. Ordinary one-way analysis of variance (ANOVA), followed by Dunnett’s multiple comparisons test. *P* value *<0.05, **<0.01, ***<0.001 and ****<0.0001 using GraphPad Prism.

To investigate whether the ER stress response is activated in the colon during infection, we infected streptomycin-pretreated mice with *S*. Typhimurium SL1344 and humanely euthanized the mice at 24, 48, and 72 h post-infection. Expression of the UPR target genes *Xbp1* and *Hspa5* is significantly increased in the colon of infected mice at all time points tested, indicating that the ER stress response is activated during infection (Fig. 1A and B). In contrast, *Chop* expression is significantly reduced at 72 h post-infection (Fig. 1C). These results suggest that ER stress is activated during *Salmonella* infection but that activation of the three UPR branches may be differentially regulated during intestinal inflammation.

### *Chop^-/-^* mice have reduced *S*. Typhimurium colonization, dissemination, and tissue pathology

**Fig 2:**
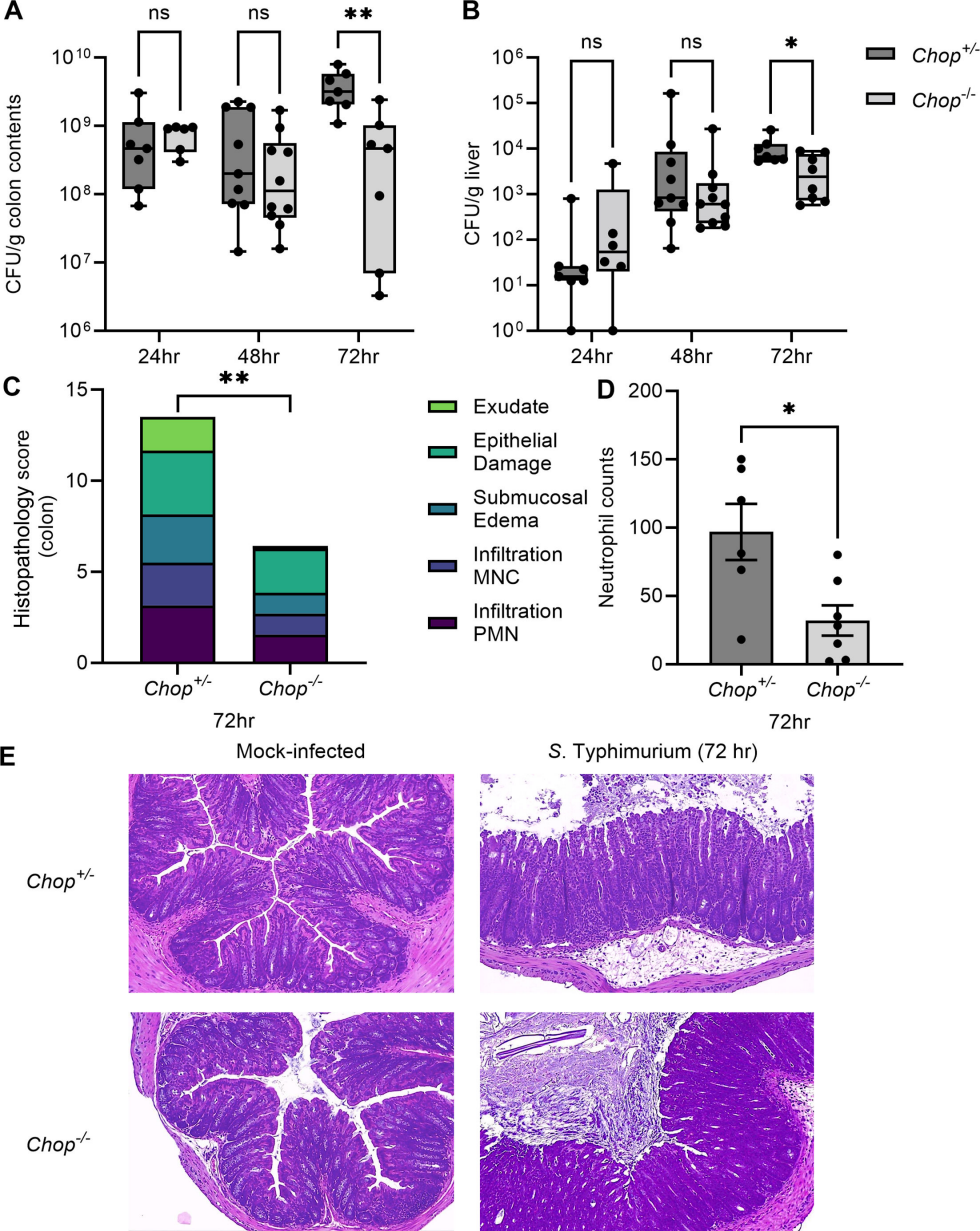
CHOP contributes to colonization, dissemination, and pathology during *S. Typhimurium* infection. Streptomycin-pretreated *Chop^-/-^* and *Chop ^+/-^* littermate control mice were infected with *S*. Typhimurium (SL1344, 10^8^ cfu/mouse), and bacterial numbers were determined in colon contents (**A**) and liver (**B**) at 24, 48, and 72 h post-infection. (**C**) Total histopathology scores for colon sections at 72 h p.i. (**D**) Total neutrophil count. (**E**) Representative images of the colon of *Chop^-/-^* and *Chop ^+/-^* mice mock-treated or infected with *S.* Typhimurium for 72 h. Data are shown as mean ± standard error of the mean or min to max, with 6–10 mice per group. (A and B) Multiple unpaired *t*-tests. (C and D) Unpaired *t*-tests. *P* value *<0.05, **<0.01, ns (not significant) using GraphPad Prism.

The dissociation between UPR activation and reduced *Chop* expression in *Salmonella*-infected colonic tissue was surprising, since it has been demonstrated previously that other bacterial pathogens induce the expression of *Chop* (23–27). To investigate the role of CHOP during *Salmonella* infections, we infected CHOP-deficient mice (*Chop^-/-^*)*,* heterozygous (*Chop^+/-^*), and wild-type (*Chop^+/+^*) littermate control mice with *S*. Typhimurium SL1344. Expression of *Hspa5* and *Xbp1* was not significantly different in *Chop^-/-^* mice compared to the control mice, indicating that ER stress is induced in CHOP-deficient mice (Fig. S2A and B). Importantly, no significant differences in colon colonization and dissemination to the liver were observed when comparing heterozygous (*Chop^+/-^*) and wild-type (*Chop^+/+^*) mice (Fig. S2C and D). We therefore continued using heterozygous *Chop ^+/-^* littermate control mice in comparison with *Chop^-/-^* mice for consecutive experiments. The *Chop^-/-^* mice, on the other hand, had significantly lower bacterial numbers in the colon and liver 72 h post-infection (Fig. 2A and B). At 72 h post-infection, the *Chop^-/-^* mice had significantly reduced histopathology scores and neutrophil counts (Fig. 2C through E). There were no significant differences in histopathology scores between the *Chop^-/-^* mice and the littermate control mice at 48 h post-infection (Fig. S3).

### CHOP contributes to the expression of pro-inflammatory cytokines during *S*. Typhimurium infection

**Fig 3:**
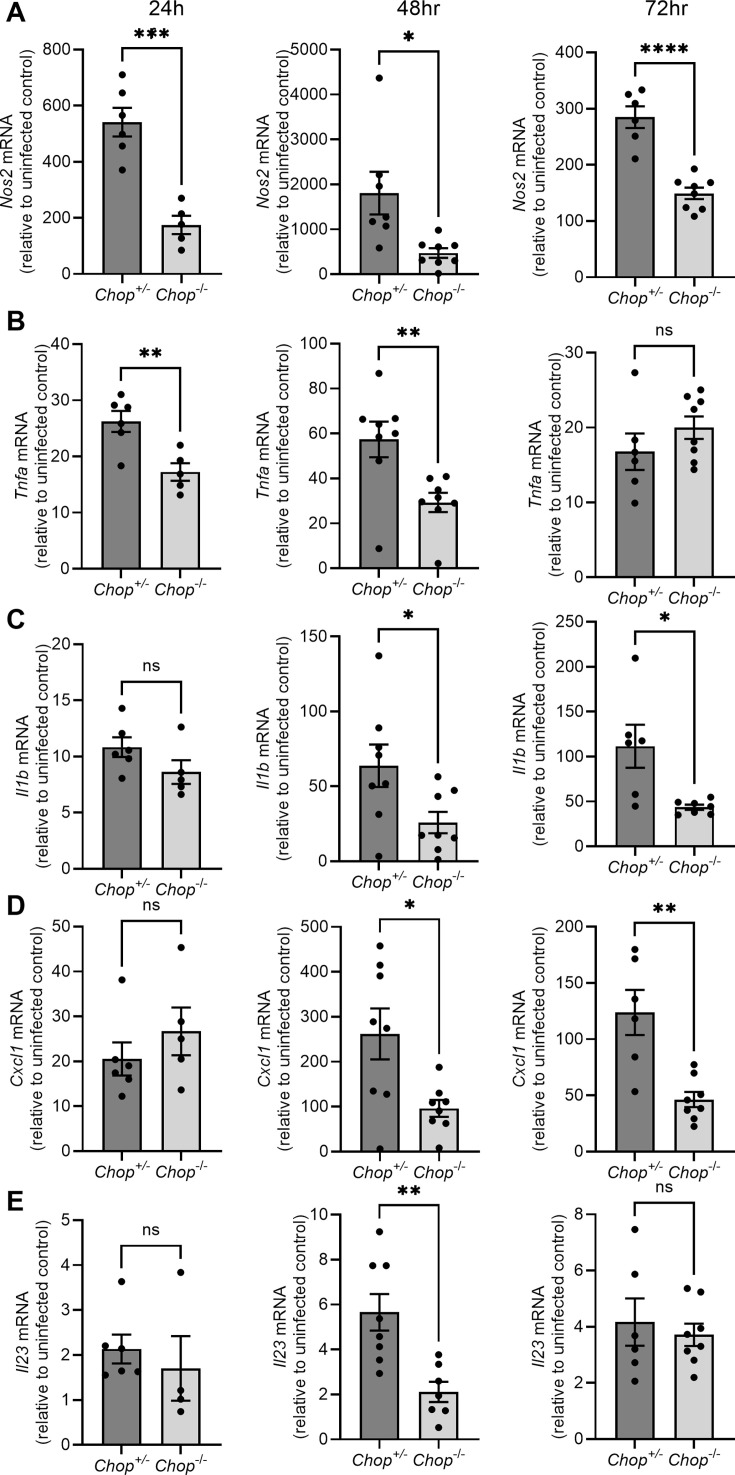
*Chop^-/-^* mice have reduced cytokine expression. RNA from the colon of infected and uninfected *Chop^-/-^* and *Chop ^+/-^* littermate control mice was extracted and analyzed by qRT-PCR to determine the levels of *Nos2* (**A**), *Tnfa* (**B**), *Il1b* (**C**), *Cxcl1* (**D**), and *Il23* (**E**) at 24, 48, and 72 h post-infection. Data are shown as mean ± standard error of the mean with 5–8 mice per group. Unpaired *t*-tests. *P*-value *<0.05, **<0.01, ***<0.001 and ****<0.0001, ns (not significant) using GraphPad Prism.

*S*. Typhimurium utilizes inflammation to drive bacterial expansion, suggesting that the reduced bacterial burdens and histopathology at 72 h p.i. may be linked to a decrease in the inflammatory response (2) (Fig. 2). At 24 h post-infection, we observed decreased expression of *Nos2* and *Tnfa* in the colon of *Chop^-/-^* mice, suggesting that CHOP has a role in the induction of inflammation (Fig. 3A and B). We did not observe any differences at the basal level in mock-infected *Chop^-/-^* mice and littermate control mice (data not shown). At the later time points, *Nos2* and *Tnfa* expression remained decreased, and *Il1b, Cxcl1*, and *Il23* were also reduced (Fig. 3). The reduced gene expression at 24 and 48 h post-infection was not the result of reduced bacterial numbers in the colon, since the difference in colon colonization was observed only at 72 h post-infection (Fig. 2A and B).

### CHOP expression in intestinal epithelial cells does not contribute to *S*. Typhimurium pathogenesis

**Fig 4:**
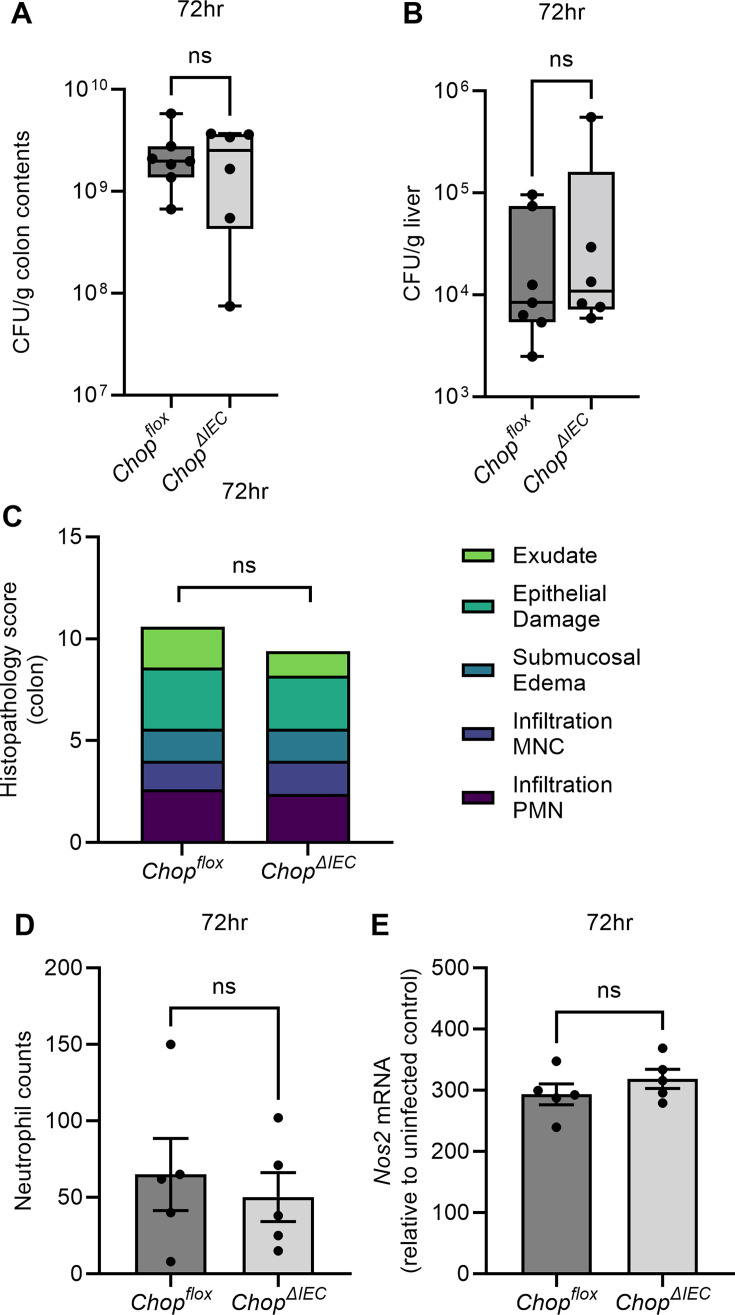
CHOP expression in IECs does not contribute to resistance to *S.* Typhimurium infections. Streptomycin-pretreated *Chop^ΔIEC^* and *Chop^flox^* littermate control mice were infected with *S*. Typhimurium (SL1344, 10^8^ cfu/mouse), and CFUs were determined in colon contents (**A**) and liver (**B**) at 72 hpi. (**C**) Total histopathology scores and (**D**) neutrophil counts in the colon at 72 hpi. (**E**) *Nos2* mRNA levels in the colon. Data are shown as mean ± standard error of the mean or min to max with 5–7 mice per group. (**A and B**) Mann-Whitney U test. (**C–E**) Unpaired *t*-tests, ns (not significant).

Previous studies with mouse models of T cell-mediated and bacteria-driven colitis have demonstrated that both CHOP mRNA and protein expression are downregulated in intestinal epithelial cells (29). To investigate whether CHOP expression in IECs results in increased *Salmonella* burden, we infected *Chop*^ΔIEC^ mice and littermate control mice (*Chop^flox^*) with *S*. Typhimurium. At 48 and 72 h post-infection, we did not observe any significant differences in colon colonization or dissemination to the liver (Fig. 4A and B; Fig. S4A and B). Moreover, no differences were observed in histopathology scores or neutrophil counts (Fig. 4C and D; Fig. S4C and D), suggesting that CHOP expression in IECs does not contribute to *Salmonella* expansion or pathology in the intestinal tract. Additionally, we did not observe significant differences in gene expression of *Nos2*, *Cxcl1*, *Il1b*, *Tnfa,* and *Il23* (Fig. 4E; Fig. S4 and S5). Altogether, this indicates that CHOP expression in IECs does not contribute to *Salmonella*-induced colitis, contrary to other colitis models (29).

**Fig 5:**
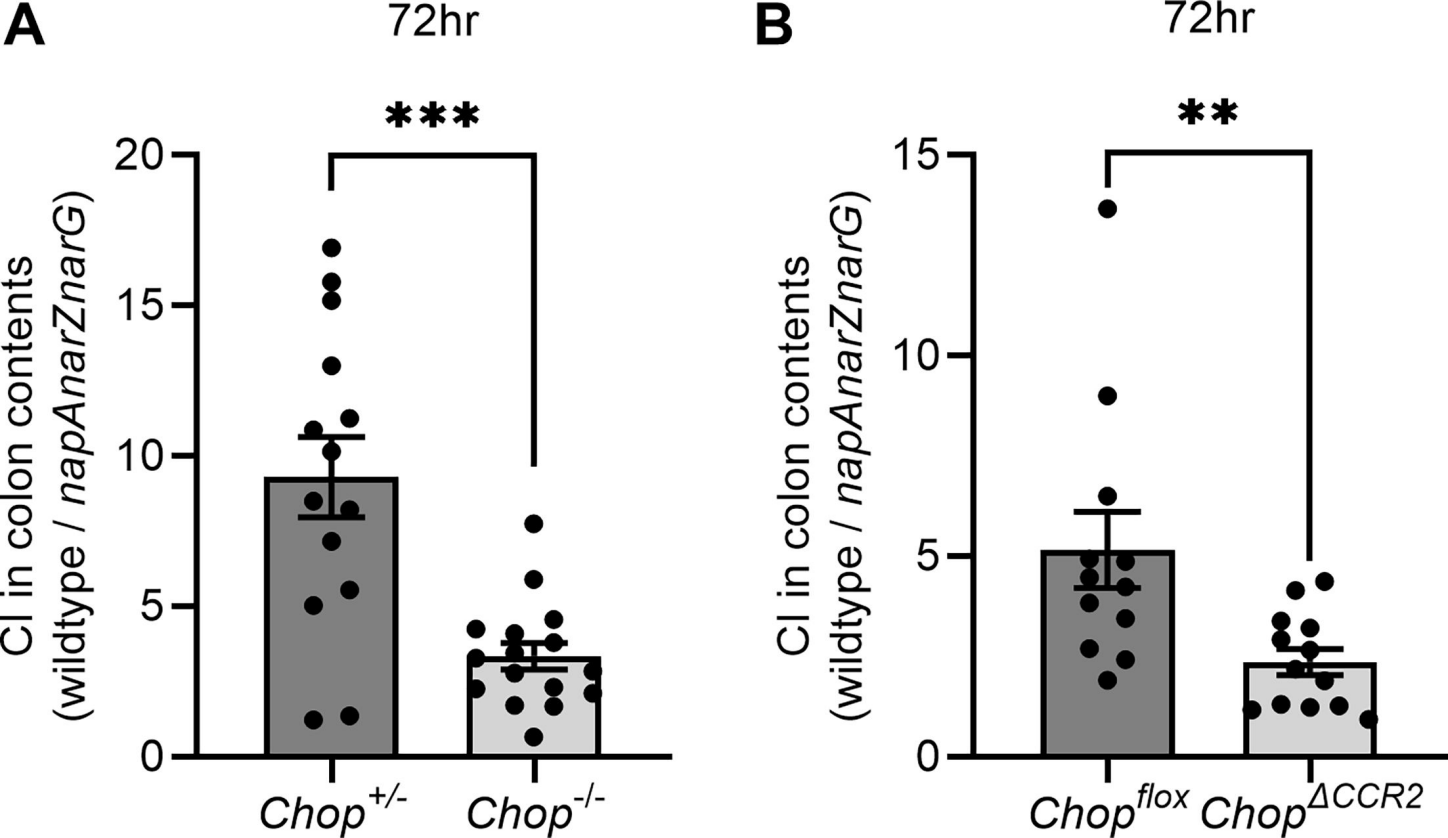
CHOP in CCR2^+^ cells contributes to nitrate production, resulting in a growth benefit for *S*. Typhimurium. Streptomycin-pretreated *Chop^-/-^* and *Chop ^+/-^* littermate control mice (**A**) and *Chop*^ΔCCR2^ and *Chop^flox^* littermate control mice (**B**) were infected with a 1:1 mixture of wild-type *S*. Typhimurium SL1344 and *ΔnapAnarZnarG*. The competitive index (CI) in the colon contents was determined at 72 h post-infection. Data are shown as mean ± SEM. Unpaired *t*-test. *P*-value **<0.01, ***<0.001 using GraphPad Prism.

### *Chop^-/-^* mice have reduced iNOS expression, leading to decreased colon colonization

*Nos2* encodes the inducible nitric oxide synthase (iNOS), which is present at lower levels in the colon of *Chop^-/-^* mice at 48 h and 72 h after infection with *S*. Typhimurium (Fig. S6A and B). The reduced expression of *Nos2* mRNA and iNOS protein is of particular interest because iNOS has been implicated in enhanced nitrate production in the intestinal lumen, thereby contributing to *S*. Typhimurium replication via nitrate respiration (16, 17). To investigate whether deletion of *Chop* would decrease the bioavailability of nitrate in the colon, *Chop^-/-^* and heterozygous control mice were infected with a 1:1 mixture of wild-type SL1344 and a nitrate respiration-deficient strain (*napAnarGnarZ*) (17). At 48 and 72 h post-infection, mice were sacrificed, and CFUs in the colon contents were enumerated. The fitness advantage conferred to the wild-type SL1344 by nitrate respiration was significantly diminished in the *Chop^-/-^* mice at 72 h, but not at 48 h post-infection (Fig. 5A; Fig. S6C). These results suggest a role for CHOP in the production of nitrate, resulting in the growth benefit for *S*. Typhimurium via nitrate respiration.

### CHOP expression in CCR2^+^ myeloid cells contributes to nitrate respiration

Given that CHOP expression in IECs does not affect *Salmonella*-induced colitis (Fig. 4), we hypothesize that CHOP expression in myeloid cells may underlie nitrate-mediated respiration and luminal expansion. CC-chemokine receptor-2 (CCR2) is critical in the migration of Ly6C^hi^ monocytes from the bone marrow, and CCR2^+^ monocytes contribute to nitrate availability for *Salmonella* in the inflamed intestine (18, 19, 30). To investigate whether CHOP expression in CCR2^+^ myeloid cells contributes to *S*. Typhimurium growth through nitrate respiration, we infected *Chop*^ΔCCR2^ and *Chop^flox^* mice with a 1:1 mixture of wild-type SL1344 and the *napAnarGnarZ* mutant (17). The fitness advantage conferred to the wild-type SL1344 by nitrate respiration was significantly reduced in the *Chop*^ΔCCR2^ mice compared to the *Chop^flox^* mice, indicating that CHOP expression in CCR2^+^ inflammatory monocytes contributes to *S*. Typhimurium growth in the intestinal lumen via nitrate respiration (Fig. 5B).

### iNOS expression in CD11b^+^ colonic cells is dependent on CHOP

**Fig 6:**
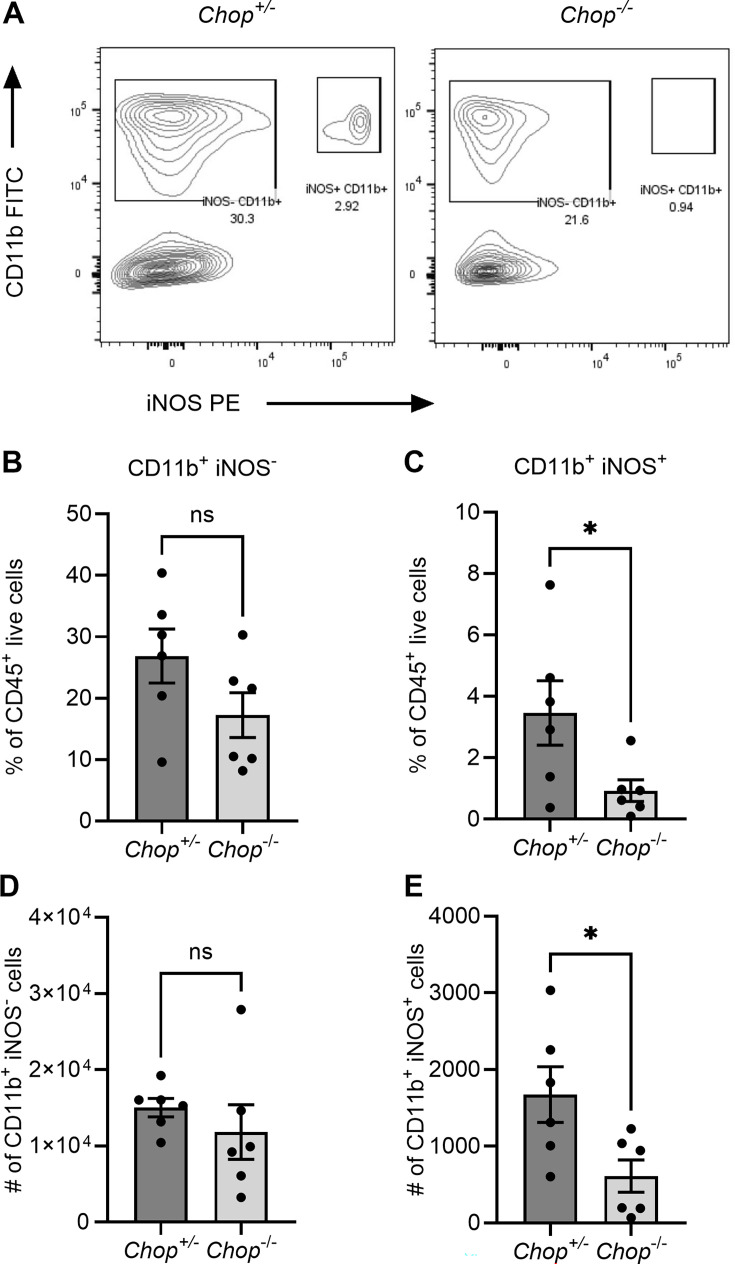
CHOP-deficient CD45^+^CD11b^+^ colonic cells produce less iNOS. Representative flow plots of staining for CD11b and iNOS on cells isolated from colonic tissue of *S*. Typhimurium-infected mice at 72 h p.i. (**A**). Percentage of CD45^+^ live cells that are CD11b^+^iNOS^-^ (**B**) and CD11b^+^iNOS^+^ (**C**). Quantification of number of CD11b^+^iNOS^-^ cells (**D**) and CD11b^+^iNOS^+^ cells (**E**). Data are shown as mean ± standard error of the mean with 6 mice per group. Unpaired *t*-tests. *P*-value *<0.05, ns (not significant) using GraphPad Prism.

To investigate whether CHOP expression in myeloid cells contributes to the production of nitrate, flow cytometric analysis was performed on live colonic cells for the expression of iNOS (Fig. 6A). Isolated live cells were gated for a population that was positive for the leukocyte marker CD45 and positive for CD11b, the marker for inflammatory phagocytes (i.e., monocytes/macrophages and neutrophils). There were no significant differences in the percentage of CD45^+^CD11b^+^iNOS^-^ cells isolated from *S*. Typhimurium-infected *Chop^-/-^* mice compared to littermate control mice (Fig. 6B), suggesting that CHOP expression is not required for recruitment of CD11b^+^ cells. Infected *Chop^-/-^* mice, however, have a lower percentage of live CD45^+^CD11b^+^ cells that were iNOS^+^ compared to infected littermate control mice, indicating that CHOP expression in CD11b^+^ cells contributes to iNOS production (Fig. 6C). Consistent with this finding, flow cytometric analysis disclosed that the total number of CD45^+^CD11b^+^iNOS^-^ cells from the colon of infected *Chop^-/-^* and littermate control mice was not significantly different, but *Chop^-/-^* mice have significantly lower numbers of CD45^+^CD11b^+^iNOS^+^ cells (Fig. 6D and E). Overall, our data demonstrate that CHOP expression in CD45^+^CD11b^+^CCR2^+^ cells contributes to iNOS production, resulting in increased nitrate availability that *S*. Typhimurium utilizes for respiration during infection.

## DISCUSSION

ER stress and activation of the UPR play major roles in the pathology and resolution of bacterial infections (26, 31–33). Our work expands on the role of ER stress during *S*. Typhimurium infection and indicates that CHOP promotes luminal expansion in the colon via nitrate respiration derived from CD45^+^CD11b^+^CCR2^+^ cells. Bel et al. demonstrated that *S*. Typhimurium infection increased CHOP expression in the small intestine, contrary to our finding of reduced *Chop* expression in colonic tissues. Activation of ER stress by *S*. Typhimurium in Paneth cells in the mouse small intestine resulted in activation of the secretory autophagy pathway, leading to increased lysozyme secretion in the lumen and increased lysozyme-mediated killing (27). Treating mice with tauroursodeoxycholic acid (TUDCA), thereby inhibiting the ER stress response, and thus CHOP expression, resulted in increased bacterial numbers in the small intestinal luminal contents and increased dissemination to the liver and spleen. TUDCA attenuates ER stress by inhibiting the dissociation of BiP from the receptors and therefore may not only inhibit CHOP expression but also the IRE1α and ATF6 pathways, which could contribute to the increased bacterial numbers (34). CHOP protein and *Chop* mRNA are downregulated in colonic tissues derived from mouse models of T cell-mediated and bacteria-driven colitis (29) and from colonic tissues from ulcerative colitis patients (35). In DSS- and TNBS-induced colitis, however, CHOP protein and *Chop* mRNA were increased (36). Why CHOP is differentially regulated in a variety of colitis and infection models requires further investigation. Downregulation of CHOP at later stages of *Salmonella* infection might be a protective host mechanism to limit inflammation and/or cell death. While CHOP upregulation may increase inflammation and stress-induced cell death that could contribute to host defense, *Salmonella* is known to exploit inflammation to promote its own expansion. Thus, the reduced expression of *Chop* in colonic tissue at 72 h post-infection may reflect a protective host response to restrain inflammation and prevent luminal outgrowth of the pathogen. This adaptive downregulation is not evident during the first 2 days of infection, likely because the host requires time to sense and fine-tune its response to the invading pathogen. In contrast, in mice with a complete loss of CHOP, CHOP-dependent pathways are absent from the onset of infection, resulting in reduced early Nos2 expression. Consequently, nitrate production is diminished over time, depriving *Salmonella* of a key respiratory nutrient and limiting bacterial expansion.

Specific deletion of *Chop* from IECs did not contribute to *S*. Typhimurium-induced colitis (Fig. 4). We observed no differences in pathology, inflammatory responses, or *Salmonella* burdens in the colon and liver of *Chop*^ΔIEC^ mice compared to littermate control mice. In contrast, mice with IEC-specific overexpression of CHOP were more susceptible to DSS-induced colitis, suggesting an important role for CHOP in intestinal epithelial cells during chemically induced gut inflammation (29). One of the hallmarks of disease in these mice was an increased number of apoptotic IECs compared to wild-type control mice, indicating an important role for CHOP-mediated cell death. Although we did not specifically test for increased IEC apoptosis, *Chop*^ΔIEC^ mice did not display increased histopathological scores, including epithelial damage, suggesting that *S*. Typhimurium-induced IEC apoptosis occurs via CHOP-independent mechanisms (37). Our findings, however, do not exclude a role for CHOP in the induction of IEC apoptosis during *Salmonella* infections, as deletion of one cell death pathway may be compensated for by other cell death pathways. It has recently been demonstrated that there is an extensive cross-talk between initiators and effectors of distinct cell death pathways and that loss of a single pathway does not significantly contribute to control of *Salmonella* but that combined deletion of multiple cell death pathways causes loss of bacterial control in mice (37).

Our results revealed that CHOP contributes to the increased expression of several pro-inflammatory mediators, including *Nos2, Cxcl1*, *Il1b*, *Tnfa,* and *Il23* (Fig. 3). At days 1 and 2 post-infection, we observed significantly reduced expression of these cytokines, but no differences in bacterial numbers in colon contents and liver, indicating that the reduced gene expression was not a consequence of reduced bacterial numbers. These differences were only observed in infected animals, as there were no significant differences in background expression of these cytokines in uninfected *Chop^-/-^* mice and littermate control mice (data not shown). Since activated macrophages/monocytic infiltrates are capable of secreting CXCL1, IL-1β, TNFα, IL-23, and expressing iNOS, and we excluded a role for IECs, we hypothesized that CHOP plays a role in myeloid cells by regulating the expression of pro-inflammatory cytokines. Our data demonstrate that CD11b^+^ cells isolated from colonic tissue of *Chop^-/-^* infected mice have reduced iNOS expression, suggesting that CD11b^+^ cells, which include macrophages/monocytic infiltrates, contribute to the luminal expansion of *S*. Typhimurium. Indeed, the role of monocyte-derived nitrate production in *S*. Typhimurium infection was previously demonstrated. McLaughlin et al. showed that mice lacking CCR2, which are unable to recruit monocytes to the intestine, have reduced *Nos2* and iNOS expression and reduced nitrate in the cecal lumen (18). More recently, Liou et al. demonstrated that *S*. Typhimurium does not respire nitrate derived from IEC but rather from inflammatory phagocytes (19). Here, we expand on these findings by demonstrating that CHOP expression in CCR2^+^ cells promotes nitrate production, thereby facilitating *S*. Typhimurium replication (Fig. 5B).

Nitrate bioavailability in the colon of *S*. Typhimurium-infected mice is not regulated by CHOP expressed in IEC, suggesting that IEC-derived nitrate does not contribute to nitrate utilization by *S*. Typhimurium, which is consistent with the findings from Liou et al. Interestingly, intestinal inflammation alters the colonic microbiota, supporting the growth of the Enterobacteriaceae family, which includes *Escherichia coli* (38). Winter et al. have demonstrated that one of the underlying mechanisms of growth of *E. coli* in the inflamed GI tract is via nitrate respiration (39), which was later confirmed to be derived from IECs and regulated by Peroxisome proliferator-activated receptor gamma (PPAR-γ) (19, 40). Although we demonstrate that CHOP expression in IECs does not contribute to nitrate respiration by *S*. Typhimurium, this does not exclude the possibility that CHOP does regulate iNOS expression in IECs and that CHOP-mediated epithelial-derived nitrate is utilized by *E. coli*. This could explain a possible mechanism of how CHOP expression in IEC contributes to disease severity in IBD patients as well as in DSS- and TNBS-induced colitis models, and why a bloom of *E. coli*, and not *S*. Typhimurium, is associated with IBD (29, 38, 39, 41).

The mechanism by which CHOP controls the expression of iNOS and a variety of cytokines remains to be elucidated. CHOP is a transcription factor and thus might be able to directly influence the transcription of cytokines (23). Alternatively, CHOP may control the expression of pro-inflammatory mediators indirectly by altering the polarity of macrophages (42, 43). Classically activated macrophages (CAM or M1 macrophages) produce high levels of pro-inflammatory cytokines such as IL-1, TNFα, and IL-23, as well as increased iNOS expression, whereas alternatively activated macrophages (AAM or M2 macrophages) are associated with attenuating inflammation, tissue repair, and wound healing (44). Consistent with this, CHOP has been shown to play a role in the polarization of adipose tissue macrophages. High-fat diet (HFD) feeding of *Chop^-/-^* mice skewed the recruitment of infiltrating macrophages more toward a M2 phenotype compared to wild-type control mice on a HFD, highlighting a role for CHOP in maintaining a pro-inflammatory macrophage program (42). Beyond macrophage polarization, CHOP has also been implicated more broadly in impaired tissue repair and epithelial cell proliferation (29), suggesting that loss of CHOP may promote more efficient tissue repair and a faster resolution of inflammation. In this context, reduced CHOP activity, or its complete absence in *Chop^-/-^* mice, may promote a more rapidly resolving inflammatory environment, facilitating restoration of epithelial integrity. Consistent with this idea, our histopathological analysis reveals that total pathology scores in *Chop^-/-^* mice are lower at 72 h compared with 48 h post-infection (Fig. 2 and 3). These findings support the notion that CHOP deficiency may enhance regenerative processes and contribute to improved tissue repair following *Salmonella* infection. At the same time, this raises the intriguing possibility that downregulation of CHOP represents a protective host response; however, this could be exploited by *Salmonella*. By suppressing CHOP expression, *Salmonella* may indirectly promote a shift toward M2 macrophages, which are metabolically different than M1 macrophages and may provide a more favorable niche for *Salmonella* for long-term persistence (45, 46).

Mechanistically, the polarization of macrophages might be mediated via CHOP repression of PPAR-γ activation. In IECs, it has been demonstrated that ER stress-induced CHOP can act as a repressor of PPAR-γ by sequestering the transcription factor C/EBPβ to prevent it from binding to the promoter region of PPAR-γ, resulting in an increased inflammatory response (47). PPAR-γ activation suppresses the immune state of macrophages by repressing transcription of inflammatory mediators, including *Nos2*, *Tnfa*, and *IL1b*. Therefore, in the absence of CHOP, increased PPAR-γ signaling allows for reduced cytokine and *Nos2* expression and subsequently reduced levels of nitrate that can be utilized by Enterobacteriaceae for anaerobic respiration (40).

In summary, our results expand on the role of CHOP in the induction of intestinal inflammation during *S*. Typhimurium infection, which increases bacterial expansion through increased nitrate bioavailability. Although the expression of the ER target genes, *Hspa5* and *Xbp1,* is increased, *Chop* mRNA expression is reduced in colonic tissues during *S*. Typhimurium infection, suggesting that downregulation of *Chop* might be regarded as a protective host response mechanism (29), since *Chop^-/-^* mice have reduced histopathology and *S*. Typhimurium colonic burden. Overall, we demonstrate that the host factor CHOP drives the production of nitrate from CCR2^+^ cells, which is utilized by *Salmonella* to expand in the intestinal tract.

## MATERIALS AND METHODS

### Mice

*Chop*^-/-^ (B6.129S(Cg)-Ddit3^tm2.1Dron^/J, strain 005530) mice and wild-type C57BL/6 mice were purchased from Jackson Laboratory. *Chop*^-/-^ mice were bred with wild-type mice to generate *Chop*^+/-^ mice, and then, *Chop*^+/-^ mice were bred with *Chop*^-/-^ mice to get *Chop*^+/-^ and *Chop*^-/-^ littermates for experiments. *Chop^floxl^* (B6.Cg-Ddit3^tm1.1Irt^/J, strain 030816) and Villin-Cre (B6.Cg-Tg(Vil1-cre)997Gum/J, strain 004586) mice were purchased from Jackson Laboratory. CCR2-Cre mice were generously provided by Andres Vazquez-Torres (University of Colorado Anschutz Medical Campus). *Chop^flox^* mice were bred with Villin-Cre and CCR2-Cre mice to generate *Chop*^ΔIEC^ and *Chop*^ΔCCR2^ mice, respectively. *Chop^flox^* mice negative for Cre were used as littermate control mice. All mice were genotyped using protocols and primers from the Jackson Laboratory.

### *S*. Typhimurium infection

Male and female mice (6–10 weeks of age) were pretreated with 20 mg of streptomycin in 100 μL of water by oral gavage 24 h before infection. Mice were infected with 10^8^–10^9^ CFU SL1344 in 100 μL LB broth or mock infected with 100 μL LB broth via oral gavage. Mice were weighed daily and sacrificed at 24, 48, or 72 h post-infection. At the time of sacrifice, liver and colon contents were collected for bacterial enumeration. Samples for enumeration were homogenized, serially diluted, and plated on plates containing the appropriate antibiotics. Portions of the colon tissue were snap frozen in liquid nitrogen for RNA and protein extraction. Sections of colon tissue were also collected for histology. For competitive index experiments, mice were infected with a 1:1 ratio of *S*. Typhimurium SL1344 Kan^r^ and *S*. Typhimurium *narG narZ napA* Kan^r^ Carb^r^ in 100 μL LB broth. Colon contents were collected for plating, and antibiotic selection was used to determine the colonization level for each strain.

### Quantitative reverse transcription-PCR

RNA was isolated from colon tissue using TRI Reagent (Molecular Research Center) according to the manufacturer’s instructions. Reverse transcription was performed using 1 μg of DNase-treated RNA (TURBO DNA-free Kit) with TaqMan Reverse Transcription Reagents (Applied Biosystems). Real-time PCR was performed using SYBR Green PCR Master Mix (Applied Biosystems) and the QuantStudio 7 Flex real-time PCR system (Applied Biosystems). Fold change in mRNA levels was calculated using the delta-delta comparative threshold cycle (Ct) method. All targets were normalized to expression levels of *Gapdh* (Table S1 shows the primer sequences used for qRT-PCR).

### Western blot analysis

For protein extraction from tissue samples, tissue pieces were snap-frozen in liquid nitrogen at the time of harvest. RIPA buffer (10 mM Tris HCl, 1 mM EDTA, 1% Triton X-100, 0.1% sodium deoxycholate, 0.1% SDS, 140 mM sodium chloride, 1 mM phenylmethylsulfonyl fluoride, and protease inhibitor cocktail [Roche]) was added to tissue samples, and samples were homogenized using a bead beater and put on ice to lyse for 1 h. The tissue lysate was centrifuged at 13,000 rpm for 15 min at 4°C, and supernatants containing the proteins were transferred to fresh tubes. Protein concentration was measured using the Pierce BCA protein assay kit according to the manufacturer’s instructions; 30 μg of each sample was run on a 10% SDS-PAGE gel for 1 h at 150 volts. Proteins were transferred to a PVDF membrane using the Bio-Rad Trans-blot Turbo Transfer System. Membranes were then blocked for 1 h with 5% milk in TBST at room temperature. Membranes were incubated with anti-iNOS (1:500) and anti-a/b Tubulin (1:5,000) (Cell Signaling Technologies) overnight at 4°C. Secondary anti-rabbit antibody was used at 1:10,000 for 1 h at room temperature. Membranes were developed using ECL Clarity (Bio-Rad) for 4 min and then imaged using a G:BOX (Syngene).

### Histopathology

Histological samples from mouse experiments were collected in 10% buffered formalin until the time they were embedded, sliced, mounted, and hematoxylin and eosin-stained by the histology core in Dr. Rubin Tuder’s laboratory at the University of Colorado Anschutz Medical Campus. All scoring was done blinded by a veterinary pathologist (Dr. Mariana X. Byndloss at Vanderbilt University). The following pathological changes were scored: (i) neutrophil infiltration, (ii) infiltration by mononuclear cells, (iii) submucosal edema, (iv) epithelial damage, and (v) inflammatory exudate. The pathological changes were scored on a scale from 0 to 4 as follows: 0, no changes; 1, detectable; 2, mild; 3, moderate; and 4, severe. Neutrophil counts were determined by high-magnification (×400) microscopy, and the numbers were averaged from 10 microscopic fields for each animal.

### Single-cell isolation

After animal sacrifice, the colon was removed and put in ice-cold RPMI medium. Colons were washed with ice-cold PBS to remove any contents. The colons were then cut longitudinally and placed in PBS containing 7.5 mM HEPES and 1 mM EDTA (IEL buffer) and shaken at 37°C for 10 min. Tissues were then filtered through a 70 μm filter (Fisher Scientific) and put in fresh IEL buffer and shaken for an additional 10 min. The samples were filtered again and minced with scissors. Tissues were then digested in RPMI supplemented with 10% FBS, 0.25% β-mercaptoethanol, 3.75 mM HEPES, and 0.54 mg/mL type IV collagenase (Worthington) for 30 min at 37°C. Digested tissue was then forced through a 70 μm filter, and single cells were counted using a C20 Automated Cell Counter (Bio-Rad). For staining, 3 × 10^6^ cells were plated in round-bottom, 96-well plates.

### Flow cytometric analysis

Single-cell suspensions were incubated with anti-CD45 (1:100, Brilliant Violet 785, Biolegend), anti-CD11b (1:50, FITC, Biolegend), and live/dead (1:1,000, eFlour 780 Fix Viability, Invitrogen) for 30 min at 4°C. After incubation, cold PBS was added to wash cells, and samples were spun down at 1,500 rpm for 5 min. For intracellular staining, the Cytofix/Cytoperm kit (BD) was used according to the manufacturer’s instructions. Briefly, fixation/permeabilization solution was added, and the cells were then incubated for 20 min at 4°C. Cells were then washed using perm/wash buffer to remove the fixation solution. Cells were then stained with anti-iNOS (1:100, PE, Invitrogen) for 30 min at 4°C and then washed using perm/wash buffer. Single-stained controls for compensation were done using anti-rat and anti-hamster Igκ/negative control beads (BD). Cell staining was analyzed using LSRFortessa X-20 cell analyzer (BD) at the CU | AMC ImmunoMicro Flow Cytometry Shared Resource. Flow analysis was then performed using FlowJo (BD).

### Statistical analysis

Data analysis was performed using GraphPad Prism. Data are shown as mean ± standard error of the mean (SEM) or min to max. One-way ANOVAs, Student’s *t*-tests, and Mann-Whitney U tests were performed. More information about statistical analysis can be found in the individual figure legends.

## SUPPLEMENTAL MATERIAL

Figure S1 mbio.01008-26-s0001.tif Schematic of the UPR.

Figure S2 mbio.01008-26-s0002.tif Activation of the UPR in Chop-deficient mice.

Figure S3 mbio.01008-26-s0003.tif Histopathology at 48 h in Chop-deficient mice.

Figure S4 mbio.01008-26-s0004.tif Infected Chop DIEC mice at 48 hpi.

Figure S5 mbio.01008-26-s0005.tif Cytokine expression in Chop DIEC mice at 72 hpi.

Figure S6 mbio.01008-26-s0006.tif Reduced iNOS expression in Chop-deficient mice.

Table S1 mbio.01008-26-s0007.docx Primers.

ASM does not own the copyrights to Supplemental Material that may be linked to, or accessed through, an article. The authors have granted ASM a non-exclusive, world-wide license to publish the Supplemental Material files. Please contact the corresponding author directly for reuse.
