# Supplementary figures and images for: Nitrate-mediated luminal expansion of Salmonella Typhimurium is dependent on the ER stress protein CHOP
Source: mBio. 2026 Jun 15;17(7):e01008-26. doi: 10.1128/mbio.01008-26 (PMC13343973; doi:10.1128/mbio.01008-26)

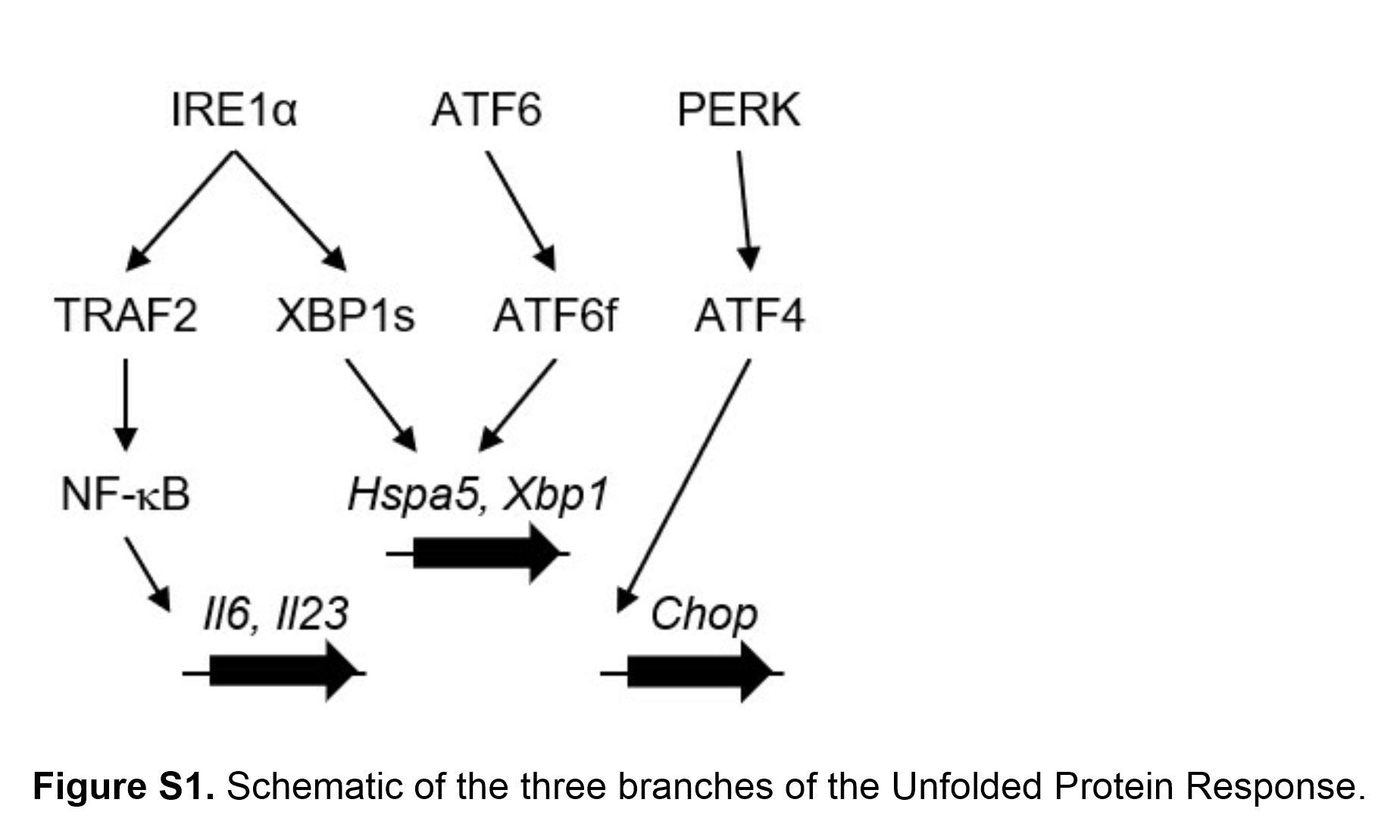

Supplement: Figure S1 — Schematic of the UPR. [file mbio.01008-26-s0001.tif]

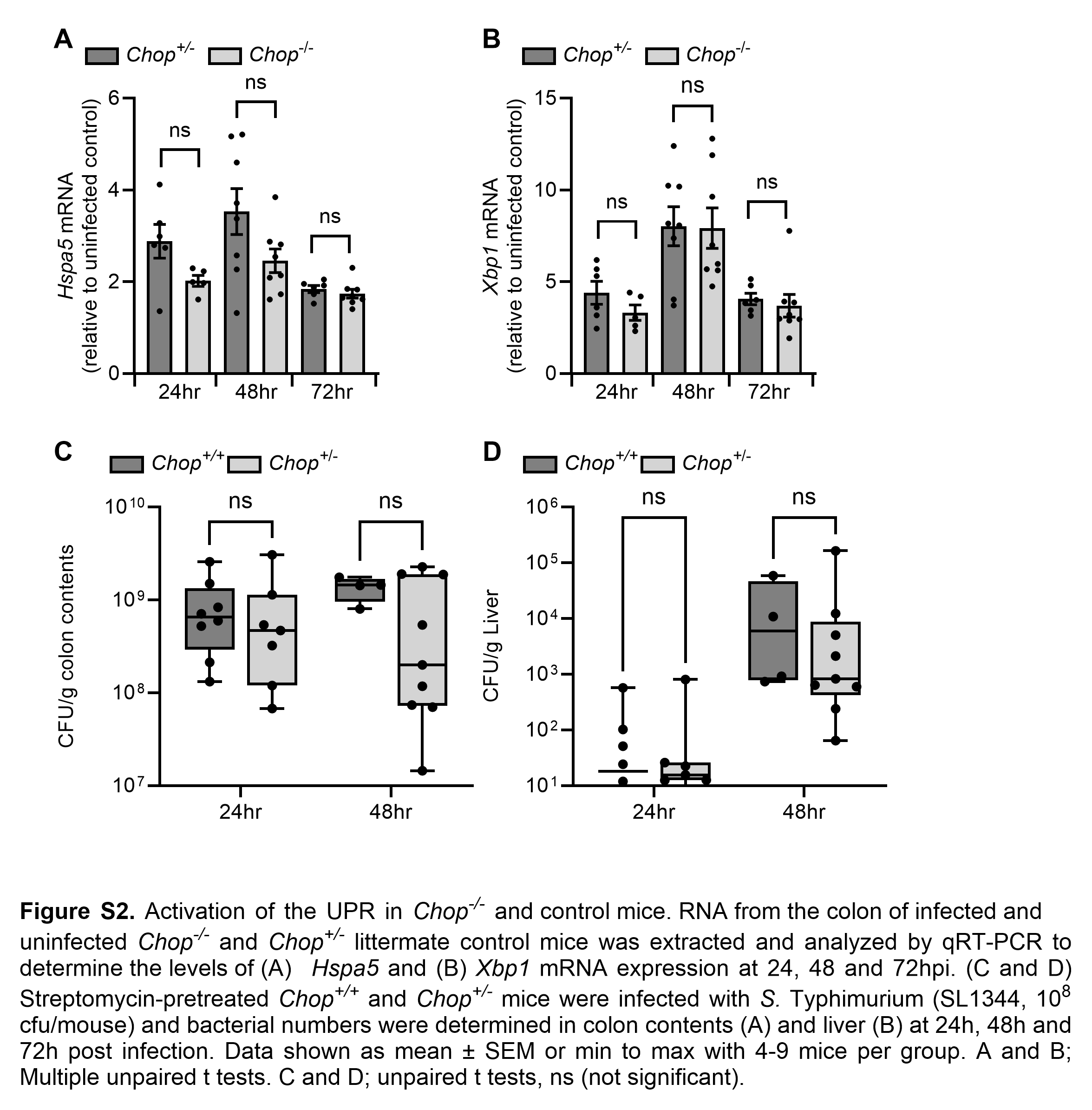

Supplement: Figure S2 — Activation of the UPR in Chop-deficient mice. [file mbio.01008-26-s0002.tif]

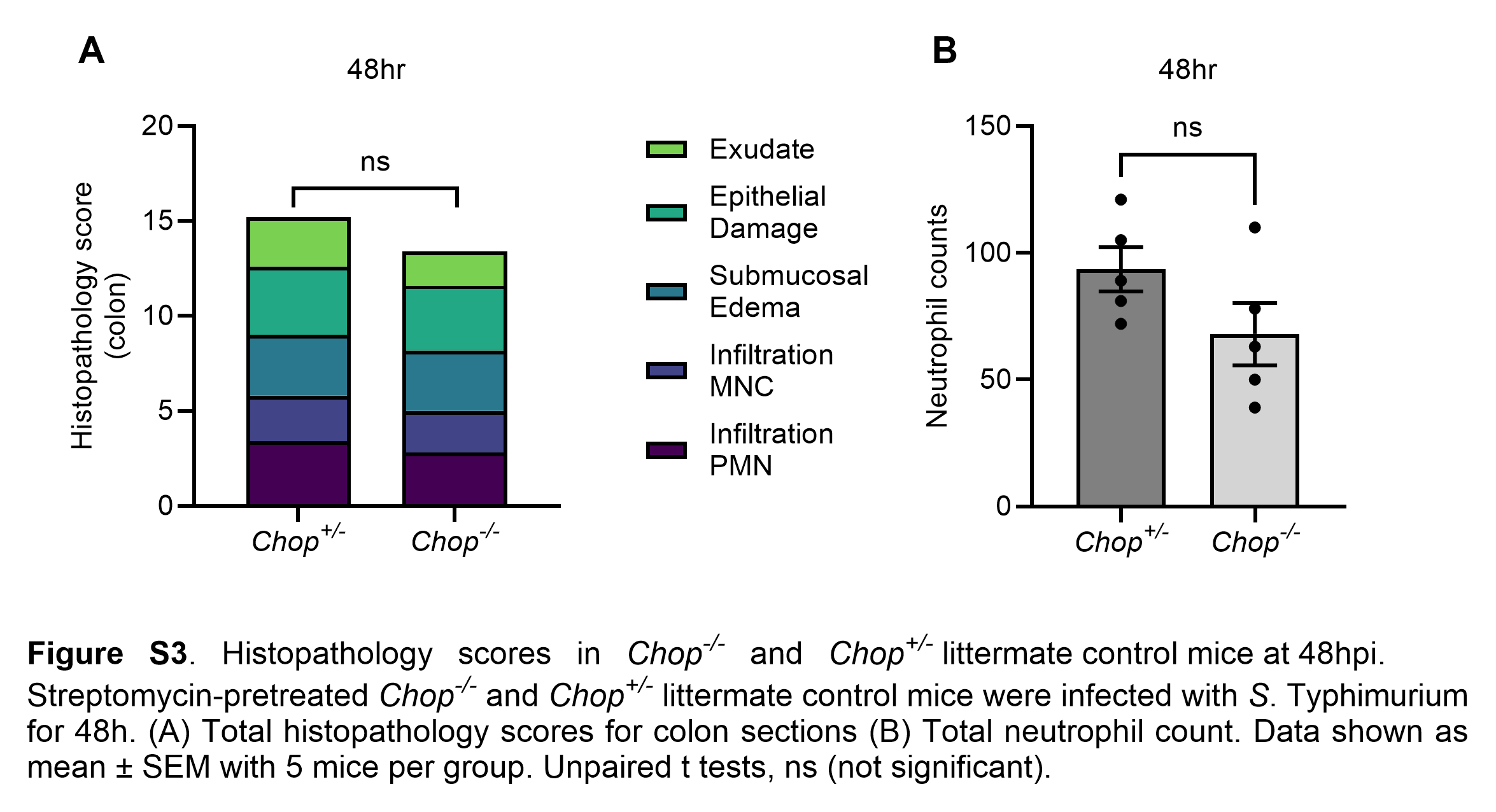

Supplement: Figure S3 — Histopathology at 48 h in Chop-deficient mice. [file mbio.01008-26-s0003.tif]

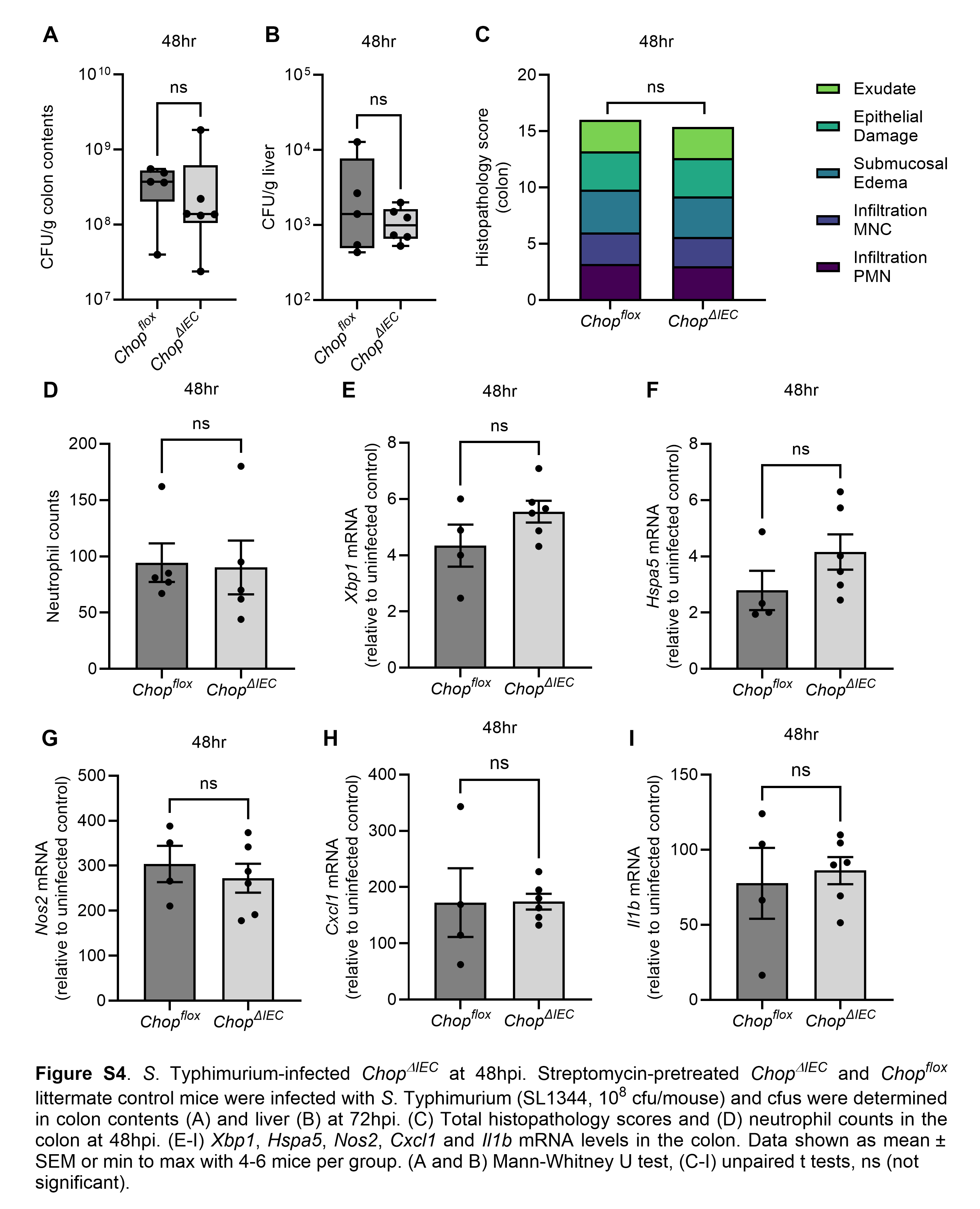

Supplement: Figure S4 — Infected Chop DIEC mice at 48 hpi. [file mbio.01008-26-s0004.tif]

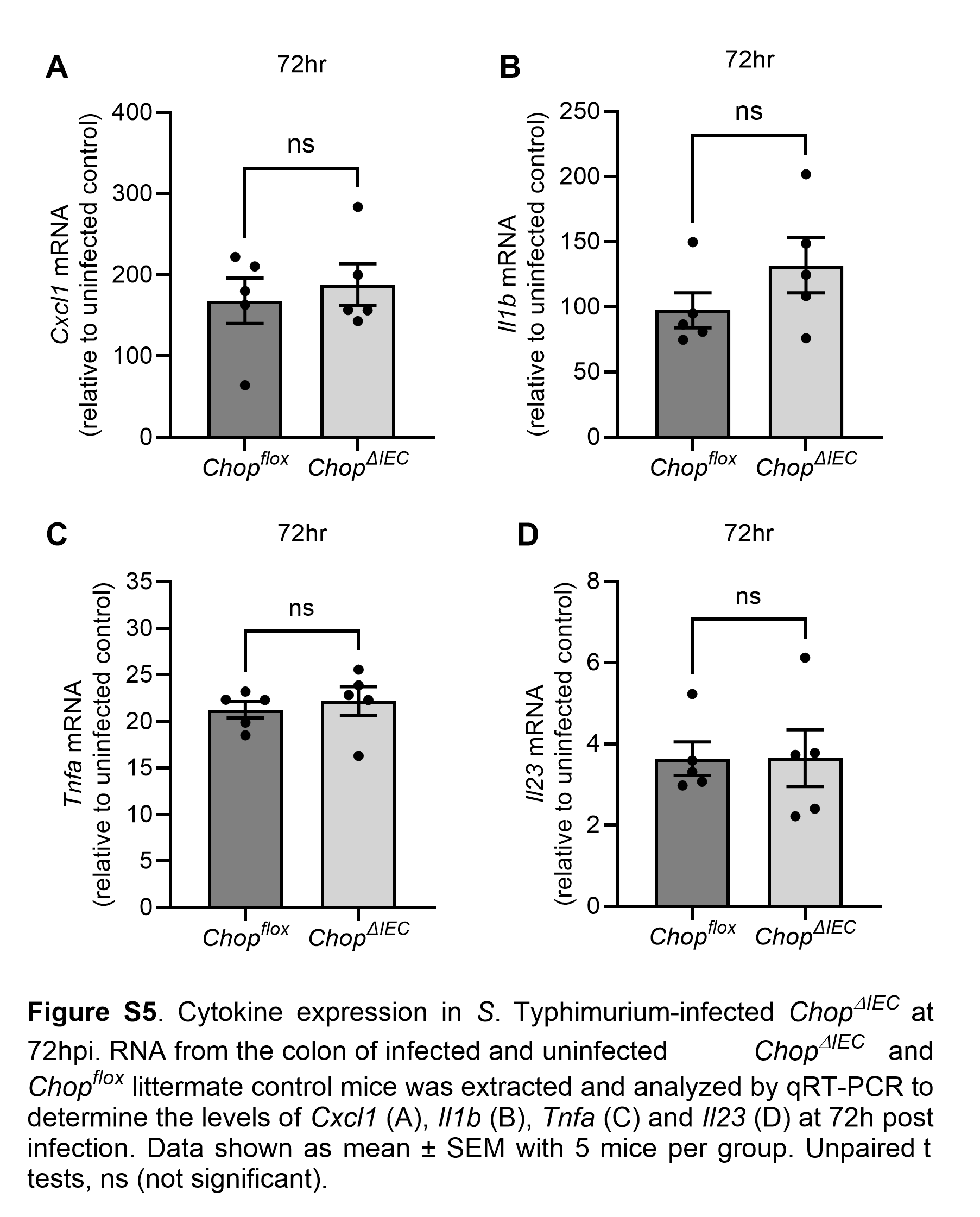

Supplement: Figure S5 — Cytokine expression in Chop DIEC mice at 72 hpi. [file mbio.01008-26-s0005.tif]

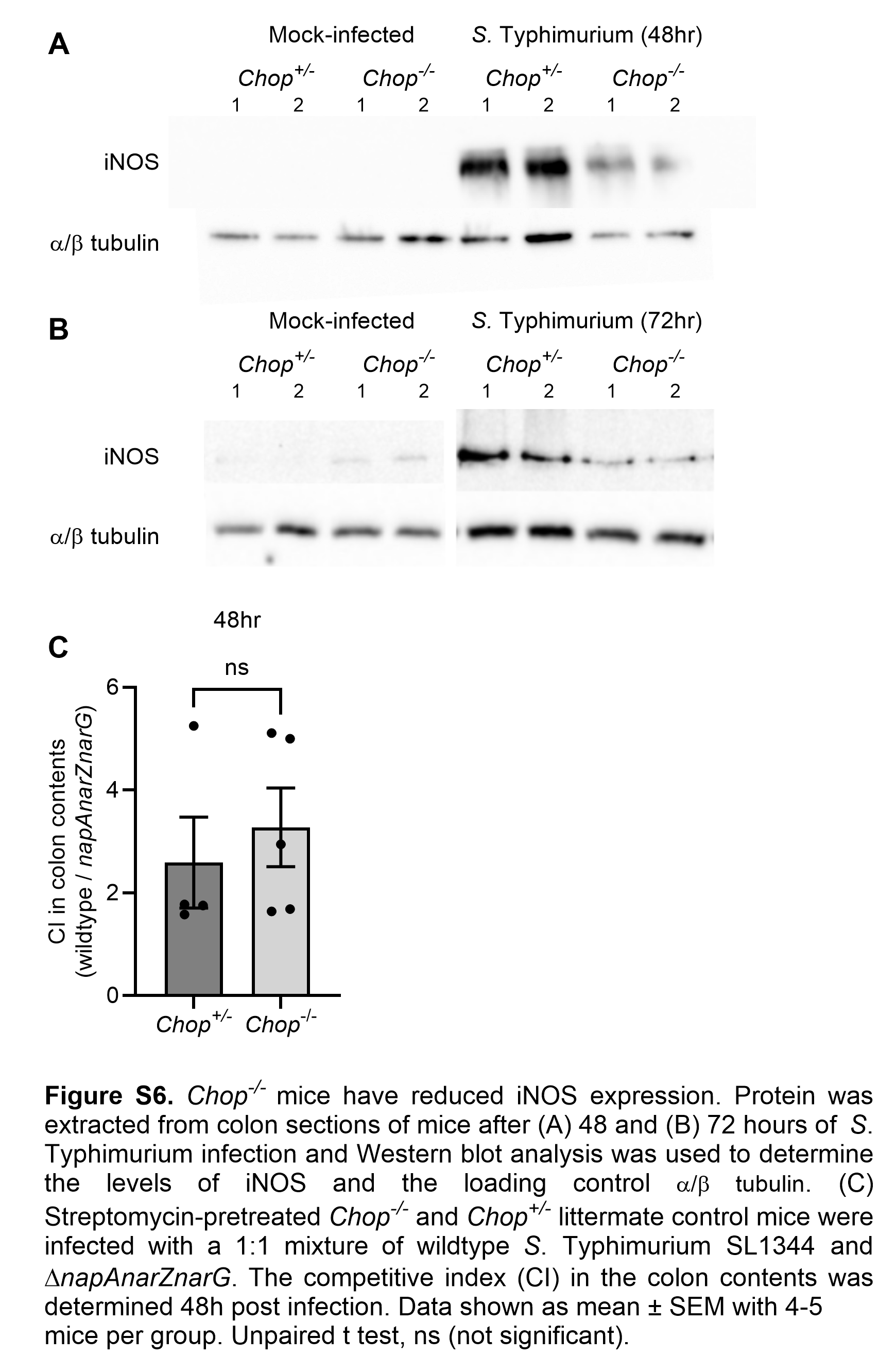

Supplement: Figure S6 — Reduced iNOS expression in Chop-deficient mice. [file mbio.01008-26-s0006.tif]
