# Supplementary material for: Nitrate-mediated luminal expansion of Salmonella Typhimurium is dependent on the ER stress protein CHOP
Source: mBio. 2026 Jun 15;17(7):e01008-26. doi: 10.1128/mbio.01008-26 (PMC13343973; doi:10.1128/mbio.01008-26)
Supplement: Table S1 — Primers. [file mbio.01008-26-s0007.docx]

**Supplementary Table 1: Primers used in this study**

| Target | Forward Sequence (5’-3’) | Reverse Sequence (5’-3’) |
| --- | --- | --- |
| *mGapdh* | TGTAGACCATGTAGTTGAGGTCA | AGGTCGGTGTGAACGGATTTG |
| *mHspa5* | GAGCGTCTGATTGGCGATGC | TTCCAAGTGCGTCCGATGAGG |
| *mXbp1* | GAGTCCGCAGCAGGTG | GTGTCAGAGTCCATGGGA |
| *mChop* | CTGGAAGCCTGGTATGAGGAT | CAGGGTCAAGAGTAGTGAAGGT |
| *mNos2* | TTGGGTCTTGTTCACTCCACGG | CCTCTTTCAGGTCACTTTGGTAGG |
| *mIl1b* | CCTGAACTCAACTGTGAAATGCC | TCTTTTGGGGTCCGTCAACTTC |
| *mCxcl1* | TGCACCCAAACCGAAGTCAT | TTGTCAGAAGCCAGCGTTCAC |
| *mIl23* | CCAGCAGCTCTCTCGGAATC | TCATATGTCCCGCTGGTGC |
| *mTnfa* | AGCCAGGAGGGAGAACAGAAAC | CCAGTGAGTGAAAGGGACAGAACC |
